# A Randomized, Double-Blind, Prospective Study to Evaluate the Effect of Oral Pregabalin in Upper Limb Surgeries Under Brachial Plexus Block

**DOI:** 10.7759/cureus.29117

**Published:** 2022-09-13

**Authors:** Brij B Kushwaha, Shailendra Singh, Vinod K Srivastava, Ravi Prakash, Reetu Verma, Sateesh Verma

**Affiliations:** 1 Anaesthesiology, King George's Medical University, Lucknow, IND; 2 Anaesthesiology and Critical Care, King George's Medical University, Lucknow, IND

**Keywords:** bupivacaine, premedication, preemptive analgesia, brachial plexus block, pregabalin

## Abstract

Context

The oral pregabalin administration preoperatively has been reported to reduce acute postoperative pain and prolong the duration of anesthesia produced by single-injection peripheral nerve blockade.

Aim

To study the effect of single dose pregabalin on duration of brachial plexus block

Settings and design

Prospective, randomised, double blind, comparative study

Material and methods

Patients were divided into two groups (groups A and B), with each group having 50 patients. In group A, the patient received a pregabalin capsule of 300 mg orally two hours before surgery with a sip of water. Group B received a placebo (vitamin B complex capsule) orally two hours before surgery. Brachial plexus block was performed, and data was collected.

Statistical analysis

Data analysis was done using SPSS version 21.0 statistical analysis software. Demographic data and clinical variables were compared using the student's t-test, chi-square test, and Mann-Whitney U test.

Results

The requirement of the first dose of analgesia was significantly earlier in group B as compared to group A (4:56±0:20 vs. 8:01±0:30 hours). Group B patients, as compared to group A patients, had significantly higher levels of pain after two hours of surgery (0.32±0.47 vs. 0.00±0.00) and at four hours of surgery (2.42±0.50 vs. 0.34±0.59).

Conclusions

Oral pregabalin prolongs analgesia from brachial plexus block without significant effect on the motor block. In addition, premedication with oral pregabalin increases the sensory block of brachial plexus block.

## Introduction

Pre-emptive analgesia is a technique in which analgesia is administered before inflicting pain so that stimulation of the pain pathway can be attenuated. The multimodal analgesia technique is utilized to block pain perception through various pathways or receptors. Systemic drugs and local nerve blocks can be utilized for the same. Brachial plexus block by supra clavicular approach is one of the most popular and reliable regional anesthesia techniques to provide anesthesia and postoperative analgesia for forearm and hand surgeries [1]. Various adjuvants have been used to prolong brachial plexus block, with the additional advantages of prolonging the duration of postoperative analgesia and reducing postoperative analgesic requirements [2]. In addition, oral pregabalin administration preoperatively has been reported to reduce acute postoperative pain [3,4] and prolong the duration of anesthesia produced by single-injection peripheral nerve blockade [5-7]. Very few studies evaluated the effect of systemic pregabalin as preemptive analgesia on the block characteristics of the brachial plexus block. This was a randomized control trial to study the effect of single-dose 300 mg oral pregabalin as premedication on the block characteristics of brachial plexus block. This study was a double-blind study in a patient undergoing elective upper limb surgery. The aim of this study was to evaluate effect of oral pregabalin in upper limb surgery under brachial plexus block with respect to characteristics of block (time to first analgesic requirement and recovery from motor block) and patient sedation.

## Materials and methods

After obtaining clearance from the Institutional Ethics committee, this randomized, double-blinded, placebo-controlled prospective study was conducted in a tertiary care hospital for a one-year duration. One hundred patients were enrolled in this study. Written and informed consent was taken from all patients and their attendants.

Inclusion criteria

Patients with American Society of Anesthesiologists (ASA) physical status of I and II, aged between 20 and 50 years, undergoing elective upper limb orthopedics surgery under brachial plexus block were included in the study.

Exclusion criteria

Exclusion criteria included patient refusal, patient switched to general anesthesia or inadequate block requiring additional sedation, any contraindication to regional anesthesia, allergic to drugs used in the study, peripheral neuropathy, BMI>32kg/m2, ASA physical status III and IV, pregnancy and lactation, and patients receiving chronic analgesic or steroid therapy.

Patients were randomly divided into two groups (groups A and B) using a computer-generated random number table, with each group having 50 patients. In group A, the patient received a pregabalin capsule of 300 mg orally two hours before surgery with a sip of water. Group B received a placebo (vitamin B complex capsule) orally two hours before surgery with a sip of water.

A detailed pre-anesthetic assessment was done in the pre-anesthetic checkup clinic. Informed and written consent was taken before enrolling patients, and the patients were also educated about the Visual Analog Scale (VAS) used for pain assessment. All patients were kept nil per oral according to standard guidelines. Patients were given an oral capsule (per the allotted group) with a sip of water two hours before the scheduled time for surgery. Then, patients were taken to the operation theatre, and venous cannulation was established, and standard monitors were attached.

The ultrasound-guided supraclavicular block was performed by an experienced anaesthesiologist, who was different from the one assessing the patient intra- and post-operatively. Both anesthesiologists were blinded to the groups.

Brachial plexus block was performed using a supraclavicular approach with ultrasound guidance under aseptic conditions. The injecting site was infiltrated with 3 ml lignocaine 2% (without preservative) subcutaneously. A nerve stimulator (InMed) was used to locate the brachial plexus. Block was given using 2 inches 22 gauzes insulated needle. The drug used for the block was 30 ml of bupivacaine 0.5%. Negative aspiration was performed after every 5 ml injection to avoid intravascular injection. A 3-minute massage was performed to facilitate even drug distribution.

After injection, the patients were assessed for sensory blockade by using a pinprick every three minutes till the onset of loss of sensation along the distribution of four nerves (median, radial, ulnar, and musculocutaneous nerves). Motor blockade was assessed every three minutes till the complete motor blockade was achieved. The contralateral upper limb was used as the control. The time of onset of the motor blockade is defined as the time between the injection and abolition of upper limb movement or ability to flex and extend only fingers, evaluated by a modified Bromage scale for the upper limb [8].

The sedation of the patient was assessed preoperatively using the Richmond sedation and agitation scale. Postoperatively, the patient was shifted to a postoperative room for monitoring and observation. All patients were given an injection of paracetamol 1 gm IV as rescue analgesia once the VAS score exceeded 3. Motor blockade levels were assessed at 1 hour, 2 hours, 4 hours, and 8 hours after surgery. VAS score was assessed at 1 hour, 2 hours, 4 hours, 8 hours, 12 hours, and 24 hours after surgery. Sedation was assessed using the Richmond agitation scale at 1 hour, 2 hours, 4 hours, 8 hours, 12 hours, and 24 hours after surgery.

The sample size was estimated by using the mean ± SD of the postoperative VAS by Cegin MB et al. [4], with 80% power at a 95% CI (two-sided), considering an alpha error of 5% and attrition bias of 10%, the final obtained sample size is 100.

The sample size formulae used are as follows: (Bernard, 5th edition)

n = (σ1*σ1+ σ2*σ2/ κ)( Z1-α/2 + Z1-β)/∆*∆

n = Sample size

σ = Standard deviation (1= 1.2 and 2=0.8)

∆ = Difference of means (0.8-0.2 = 0.6)

 κ = Ratio

Z1-α/2 = Two-sided Z value Z1-β = Power

Zi-o/2 = Two-sided Z value Zi_p = Power

Data analysis was done using SPSS version 21.0 statistical analysis software. Demographic data and clinical variables were compared using the student's t-test, chi-square test, and Mann-Whitney U test. P-value <0.05 was considered statistically significant.

## Results

Out of 100 patients enrolled in the study, 50 (50.0%) were given pregabalin orally two hours before the surgery. These patients were identified as group A; the rest 50 (50.0%) were given vitamin B complex capsule before two hours of surgery and were identified as group B.

The age of patients enrolled in the study ranged from 20 to 55 years, and the mean age was 36.97±8.73 years. However, group B patients were older than group A (39.22±6.66 vs. 36.68±9.89 years), yet this difference was not statistically significant.

The majority of patients in group A (58.0%) and group B (70.0%) were male, and the rest were female. However, the proportion of females was higher in group A compared to group B (42.0% vs. 30.0%), yet this difference was not statistically significant.

The body weight range of patients enrolled in the study was 45-65 kg. The mean body weight was 55.40±5.04 kg. The difference in the body weight of patients of group A (54.86±5.49 kg) and those of group B (55.94±4.54 kg) was not statistically significant.

Out of 100 patients enrolled in the study, 94.0% were ASA Grade II, rest 6.0% patients were ASA Grade I. ASA Grade I patients in Group A and Group B were 8.0% and 4.0%, respectively. The difference in the ASA grade of patients of the above two groups was not statistically significant. In all the patients, paracetamol was used as an analgesic drug. The amount of analgesic drug was 1 gm IV. Additional intraoperative analgesia was required in none of the subjects.

Although the duration of surgery of patients of group A (2:21±0:51 hours) was longer compared to that of group B (2:05±0:37 minutes), this difference was not statistically significant.

Also, VAS was significantly lower in group A at 4 and 8 hours (Table 1). For all the patients, irrespective of the above two groups, the grade of motor block remained at three at 1 hour and 2 hours (Table 2).

**Table 1:** Group comparison of post-operative pain (VAS score) at different time intervals.

Serial No.	Time	Group A (n=50)			Group B (n=50)			Mann-Whitney U test		
		Median	Mean	SD	Median	Mean	SD	Z	‘P’	
1-	lh	0.00	0.00	0.00	0.00	0.00	0.00	0.000	1.000	
2-	2h	0.00	0.00	0.00	0.00	0.32	0.47	-4.342	<0.001	
3-	4h	0.00	0.34	0.59	2.00	2.42	0.50	-8.719	<0.001	
4-	8h	3.00	2.86	0.45	3.00	2.98	0.59	-1.077	0.282	
5-	12h	3.00	3.14	0.50	3.00	3.18	0.52	-0.413	0.680	
6-	24h	3.00	2.68	0.65	2.00	2.46	0.58	-1.728	0.084	

**Table 2:** Group comparison of motor block.

Serial No.	Time	Modified Bromage	Total (N=100)	Group A (n=50)		Group B (n=50)		Mann-Whitney U test	
				No.	%	No.	%	Z	‘P’
1-	1h	3	100	50	100.0	50	100.0	0.000	1.000
2-	2h	3	100	50	100.0	50	100.0	0.000	1.000
3-	4h	2	67	35	70.0	32	64.0	-0.635	0.526
		1	33	15	30.0	18	36.0		
4-	8h	0	100	50	100.0	50	100.0	0.000	1.000

The range of time of the first dose of analgesia after induction in group A was 6:45-8:45 hours, while that in group B was 4:15-5:45 hours. The requirement of first dose of analgesia was significantly earlier in group B as compared to group A (4:56±0:20 vs. 8:01±0:30 hours) (Table 3).

**Table 3:** Group comparison of time of analgesic requirement after induction (in hours).

Group	No. of patients	Minimum	Maximum	Mean	SD
Group A	50	6:45	8:45	8:01	0:30
Group B	50	2:15	6:45	4:56	0:20
Total	100	2:15	8:45	6:28	1:36

At 4h, the majority of patients in both groups had grade 2 of motor block (70.0% vs. 64.0%), rest of the patients had a grade 1 of motor block. The difference in the grade of the motor block of patients in the above two groups was not found to be statistically significant. At 8h, no motor blockade was observed in any of the patients. Patients of the above two groups did not show any significant difference in any of the periods of observation.

**Figure 1:**
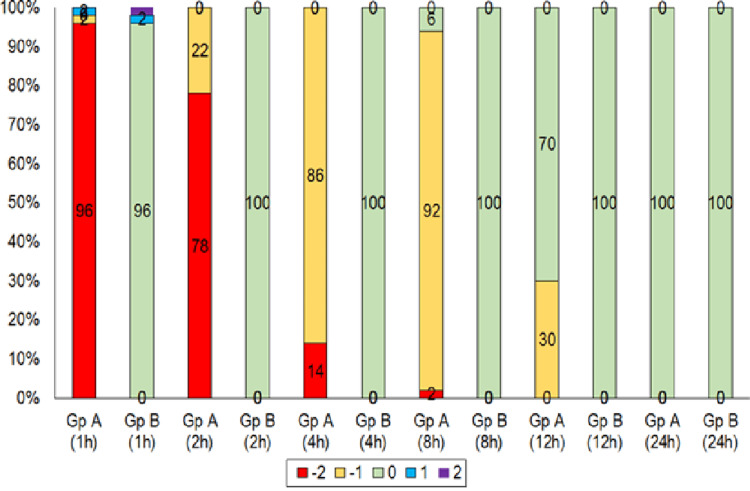
Group comparison of sedation levels.

During 1st and 2nd hours, most of the patients in group A had a -2 sedation score (light sedation), whereas group B had a 0 sedation score (alert and calm). During the 4th and 8th hours, most patients in group A had a -1 sedation score (drowsy), and group B had a 0 sedation score. During 12 hours, 30% of patients in group A had a sedation score of -1 (drowsy), and the rest, 70%, had a 0 score, whereas group B had a 0 sedation score. All the patients enrolled in the study had sedation level 0 at 24h. Significant differences in patient sedation levels were observed during all the periods except at 24 h (Figure 1).

**Figure 2:**
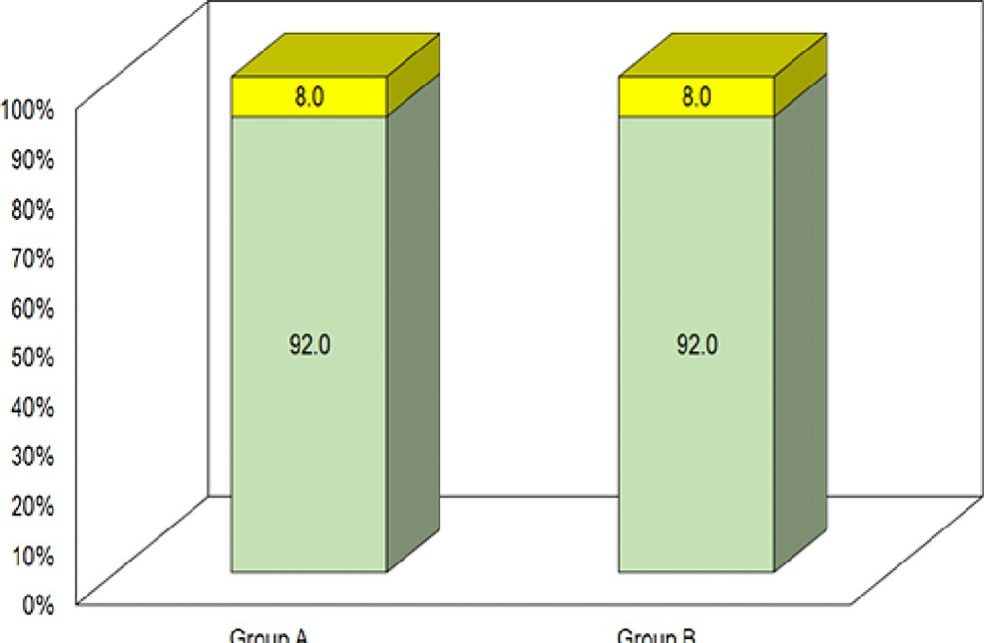
Comparison of side effects.

Complaint of nausea and vomiting was observed in an equal number of patients of the above two groups (n=4; 8.0%). No other side effect/complaint was observed in any of the patients (Table 4 and Figure 2).

**Table 4:** Group comparison of side effects.

Serial No.	Side effects	No.	%	No.	%	No.	%	%
1-	Nil	46	92	46	92	46	92	92
2-	Nausea and vomiting	4	8	4	8	4	8	

## Discussion

Brachial plexus block is popularly and widely employed in regional anesthesia for upper limb surgeries. It prevents the unwanted effect of anesthetic drugs used during general anesthesia, their complications, and the stress of the laryngoscopy and endotracheal intubation.

Post-operative pain is an actively researched and most common clinical problem in the hospital. Post-operative pain has both neuropathic and inflammatory components. Opioids are commonly used for post-operative analgesia, but their use is associated with many adverse effects and should be limited. Various drugs such as opioids, local anesthetics, nonsteroidal anti-inflammatory drugs (NSAIDs), COX-2 inhibitors, GABA analog, clonidine, and dexmedetomidine have been used as preemptive analgesia. In our study, we administered 300 mg oral pregabalin two hours before the surgery in the patients undergoing upper limb surgeries under brachial plexus block.

This prospective, randomized, double-blind clinical study was done to evaluate the effect of oral pregabalin in upper limb surgery under brachial plexus block in terms of block characteristics and patient sedation.

We observed that the motor blockade effect in both groups showed no significant difference during any observation period. In all the patients, irrespective of their group, at 1 and 3 hours, the modified Bromage score was 3. At 4 hours, it was 2 in 70% of patients of group A and 64% of patients of Group B. At 8 hours, no motor blockade was observed in any of the patients.

Our results are supported by the study conducted by Cegin MB et al. In their study, they administered oral pregabalin 75 mg (group P75), 150 mg (group P150), 300 mg (group P300), and control (group C), preemptively 1 hour before performing the USG-guided infraclavicular block for upper limb orthopedics surgery. They observed that there was no significant difference in terms of motor blockade termination in any of the groups (p value>0.05) [4].

In another study conducted by Nema N et al. (2014) [7], they evaluated the effect of the addition of 1 ml dexmedetomidine to 29 ml of ropivacaine 0.75% in brachial plexus block through the supraclavicular route in upper limb surgeries. They found that the average duration of the motor blockade in the group with the dexmedetomidine plus ropivacaine was significantly more (390.47±107.868] minutes) as compared to that with ropivacaine (278.50±66.88 minutes).

In another study conducted by Patil KN and Singh ND (2015) [9], they used clonidine as an adjuvant to ropivacaine-induced supraclavicular block. In group 1, they used 30 ml 0.75% ropivacaine and in group 2, 1 mcg/kg clonidine diluted in 1 ml normal saline with 0.75% ropivacaine. They observed that the duration of motor blockade was significantly increased in group 2 as compared to group 1 (p-value<0.001), which can be because of alpha-2 adrenoreceptor-mediated vasoconstriction and because clonidine enhances the sodium channel blocking action of local anesthetic by opening potassium channel, resulting in hyperpolarization, during which cells become unresponsive to excitatory input.

In our study, we also found that the duration of the post-operative analgesia in group A (oral pregabalin 300 mg) was significantly higher than the placebo group B (p-value <0.001). Furthermore, group A patients as compared to group B, had a significantly lower level of pain after 2 hours of surgery (0.00±0.00 vs. 0.32±0.47) and at 4 hours after surgery (0.34±0.59 vs. 2.24±0.50). Also, the time of analgesia requirement in group A was significantly more as compared to group B (p-value<0.001). The range of time of the first dose of analgesia after induction in group A was 8:01±0:30 hours, whereas that in group B was 4:56±0:20 hours.

This result was supported by the study conducted by Park M et al. (2016) [10]. Their study administered oral pregabalin 150 mg in group P and placebo in group C patients two hours before spinal anesthesia in patients undergoing urogenital surgeries. They found that post-operative 6- and 24-hour VAS were decreased in group P. According to their study, pain score at 6 hours post-operative in the P group was 3.1±1.2, whereas in group C, it was 4.2±1.0 (p-value 0.002) and at 24-hour post¬operative, for group P it was 1.6±0.7, and for group C, it was 2.2±0.7 (p-value 0.005). They also found that the time to the first request for post-operative analgesic was delayed, and lower rescue analgesia requirement was noticed during the early post¬operative 24 hours.

Bafna U et al. (2014) [11] compared the time to the first analgesia request for the group of single-dose 600 mg gabapentin and 150 mg pregabalin premedication in gynecological surgeries under spinal anesthesia. Both medications prolonged the mean duration of effective analgesia of a spinal bupivacaine block, but pregabalin showed a significantly longer duration of analgesia. In the group with pregabalin, the analgesic effect was maintained for 535.16 ± 32.86 minutes (p-value<0.001).

In another study conducted by Sebastian B et al. (2016) [12], they compared the oral pregabalin 150 mg (group P) preemptively with placebo (group C) for post-operative pain control in patients undergoing elective lower limb orthopedic surgery under spinal anesthesia and found that majority of the patients of group P had post-operative pain (VAS>3) only two times in 24 post-operative hours whereas VAS>3 was for three times in group C patients showing that group P patients had much effective post-operative pain control.

In another study by Balaban F et al. (2012) [13], they assigned patients to three different groups, placebo (group 1), pregabalin 150 mg (group 2), and pregabalin 300 mg (group 3). They came up with the results that there was a significant decrease in the VAS scores of group 2 (10.8±15.6) and group 3 (8.3±16.5) at time 0 in comparison to group 1 (37.5±19.4). They also found that at 15, 30, 60, and 120 minutes there was a significant decrease in VAS score in group 3 as compared to group 1 (p- value<0.001). VAS scores in group 3 compared with group 2 were significantly lower at 30 and 60 minutes in the post-operative period. Also, the total fentanyl consumption in group 1 was higher than in groups 2 and 3.

In our study, we also compared the sedation level of the patients in group A versus group B using the Richmond sedation and agitation scales and found that, up to 8 hours post-operative period, most of the patients in group A had sedation, whereas none of the patients in group B had sedation at any period of observation.

This result was supported by a study conducted by Kohli M et al. [6], in which they observed that Ramsay sedation scores were significantly higher in the 300 mg pregabalin group (group 3) as compared to the placebo group (group 1) at 15 minutes post operatively. In addition, Aldrete scores in group 3 were statistically significantly higher than group 1 patients at 60 minutes post-operatively.

In another study conducted by Sebastian B et al. [12], they compared the sedation and patient satisfaction scores in patients given oral pregabalin 150 mg one hour before spinal anesthesia (group P) and patients given empty capsules (group C). They found that there were increased sedation scores in group P patients in terms of the proportion of patients having a higher Ramsay sedation score. In this study, we administered preemptive oral pregabalin 300 mg two hours prior to the upper limb orthopedics surgeries under brachial plexus block with 30 ml of 0.5% bupivacaine using a peripheral nerve stimulator. We concluded that preemptive pregabalin prolongs post-operative analgesia significantly and reduces the requirement of rescue analgesia. However, preemptive pregabalin administration does not prolong the duration of motor blockade of the brachial plexus block. Therefore, preemptive pregabalin can be an effective tool for anesthesiologists in the management of post-operative pain.

## Conclusions

Preemptive oral pregabalin prolongs the sensory block of the brachial plexus block and provides longer post-operative analgesia, as evident by an increase in time for the first analgesic requirement. It also provides sedation to the patient but has no effect on the motor block. So, we conclude that oral pregabalin has a favorable effect on brachial plexus block in upper limb surgeries. Technical expertise to perform block remains the limitation of the study as an inadequate block has been excluded from the study.
